# Nuclear hormone receptor co-repressors: Structure and function

**DOI:** 10.1016/j.mce.2011.08.033

**Published:** 2012-01-30

**Authors:** Peter J. Watson, Louise Fairall, John W.R. Schwabe

**Affiliations:** Henry Wellcome Laboratories of Structural Biology, Department of Biochemistry, University of Leicester, Leicester LE1 9HN, UK

**Keywords:** HDAC, histone deacetylase, HID, histone interaction domain, LBD, ligand binding domain, ID, interaction domain, RRM, RNA recognition motif, Nuclear receptor, Co-repressor, SMRT/NCoR, TBL1, GPS2, HDAC

## Abstract

Co-repressor proteins, such as SMRT and NCoR, mediate the repressive activity of unliganded nuclear receptors and other transcription factors. They appear to act as intrinsically disordered “hub proteins” that integrate the activities of a range of transcription factors with a number of histone modifying enzymes. Although these co-repressor proteins are challenging targets for structural studies due to their largely unstructured character, a number of structures have recently been determined of co-repressor interaction regions in complex with their interacting partners. These have yielded considerable insight into the mechanism of assembly of these complexes, the structural basis for the specificity of the interactions and also open opportunities for targeting these interactions therapeutically.

## Highlights

► Co-repressors SMRT/NCoR act as “hub proteins”. ► SMRT/NCoR integrate transcription factor and histone modifying enzyme signaling. ► Structures of co-repressor complexes. ► Structural studies provide mechanistic, and potentially therapeutic, insights.

## 1 Introduction

The regulation of gene expression by nuclear receptors plays an essential role in the regulation of growth, development and homeostasis. The nuclear receptor family comprises 48 receptors in humans, and includes receptors for which the ligand is known, adopted orphan receptors and orphan receptors for which the ligand remains as yet unknown (Mangelsdorf et al., 1995; Willson and Moore, 2002). Nuclear receptors interact with a wide family of co-regulator molecules (co-activators and co-repressors). Co-activators are generally recruited to ligand bound nuclear receptors and enhance gene expression. Co-repressors fulfill the opposite role and mainly bind to un-liganded nuclear receptors and repress transcription. Co-repressors may also play a role in “resetting” chromatin following rounds of activated transcription (Wang et al., 2009).

**Fig. 1:**
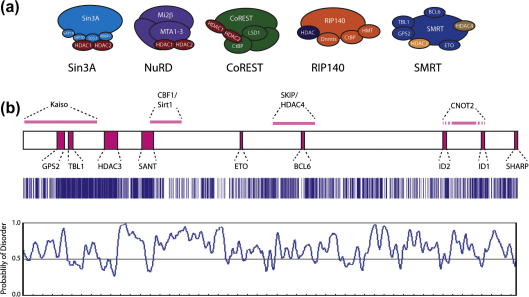
The co-repressor SMRT is mostly intrinsically disordered and acts as a platform for the interaction of many proteins. (a) Schematic diagrams of histone deacetylase containing co-repressor complexes. HDAC1 and 2 are located together in three main co-repressor complexes: Sin3A, Nucleosome Remodeling and Deacetylase (NuRD) and CoREST complexes. HDAC3 exists in a stoichiometric complex with SMRT. HDAC4 also forms part of the SMRT complex. (b) Linear representation of SMRT showing the sites of interaction of various interacting partners. Interaction sites for which there is structural data available are represented by pink boxes on SMRT, interactions for which defined amino acid boundaries have been reported are indicated by solid pink boxes, interactions which are reported but no defined boundaries are available are indicated by dashed pink boxes. The sites of interaction are drawn to scale with respect to SMRT. Sequence alignment between SMRT and NCoR, blue lines show homology, the co-repression domain can be seen as the area of high homology towards the N-terminus. A disorder prediction of SMRT (generated from RONN), showing that SMRT is predicted to be mostly disordered. Increasing probability of disorder is indicated above the line, ordered regions are indicated below the line.

Two of the best studied of the nuclear receptor co-repressors are the homologous proteins SMRT and NCoR, that were first identified through their interaction with nuclear receptors in the absence of a ligand (Hörlein et al., 1995; Chen and Evans, 1995). SMRT and NCoR also interact with many other transcription factors including: BCL6, Kaiso, ETO, MEF2C, CNOT2 and CBF1 (Ahmad et al., 2003; Gelmetti et al., 1998; Jayne et al., 2006; Kao et al., 1998; Wu et al., 2001; Yoon et al., 2003; Lutterbach et al., 1998) (Fig. 1a). SMRT and NCoR have been purified from HeLa cell extracts by several groups and have been found to form large complexes with an apparent molecular weight of between one and two megadaltons (Guenther et al., 2000; Li et al., 2000; Wen et al., 2000). Repression is mediated by recruiting multiple histone deacetylase enzymes such as HDAC1 (Ariyoshi and Schwabe, 2003; Heinzel et al., 1997; Nagy et al., 1997), HDAC7 (Kao et al., 2000), HDAC4 (Fischle et al., 2002; Huang et al., 2000), HDAC3 (Guenther et al., 2000; Li et al., 2000) and Sirt1 (Picard et al., 2004). The relative importance of each of these enzymes has yet to be fully established; however, it has been clearly demonstrated that HDAC3 recruitment to the complex is essential for repression by the thyroid hormone receptor (Ishizuka and Lazar, 2003).

## 2 Overall characteristics of SMRT/NCoR

SMRT and NCoR are large, homologous proteins (ca. 2500 aa) with an overall sequence identity of 40% (Fig. 1b). Analysis of the pattern of conservation between human SMRT and NCoR shows that there are regions of high conservation separated by regions of much lower conservation. The largest region of high conservation spans a stretch of ∼300 amino acids with 83% identity between the two proteins. Other regions of high conservation are smaller and generally span between 20 and 50 amino acids (Fig. 1b).

Predictions of secondary structure and of intrinsic disorder suggest that there are only a few regions that possess an intrinsically folded structure. Two of the regions that are predicted to be structured are proposed to fold into SANT-like domains (Aasland et al., 1996). The first of the SANT-like domains, whose structure is described below, has been shown to both recruit and activate HDAC3 and has been termed the deacetylase activation domain (DAD) (Codina et al., 2005; Guenther et al., 2001; Li et al., 2002; Zhang et al., 2002). The second SANT-like domain has been reported to interact directly with histone tails (the enzymatic substrate of HDAC3) and has been termed the histone interaction domain (HID) (Hartman et al., 2005; Yu et al., 2003). An overall picture is emerging in which SMRT and NCoR are largely unstructured platform proteins that act as a scaffold upon which the enzymatic machinery of the repression complex is built.

The largely intrinsically disordered nature of SMRT and NCoR, as well as other transcriptional co-regulators, seems to be a characteristic feature of these proteins and such properties are often associated with so-called hub proteins that have many interaction partners (Singh et al., 2007; Haynes et al., 2006). The characteristic of intrinsic disorder may reflect the need to make many highly specific but relatively low affinity interactions (due to the entropic cost of forming complexes).

## 3 Interaction of SMRT/NCoR with unliganded nuclear receptors

**Fig. 2:**
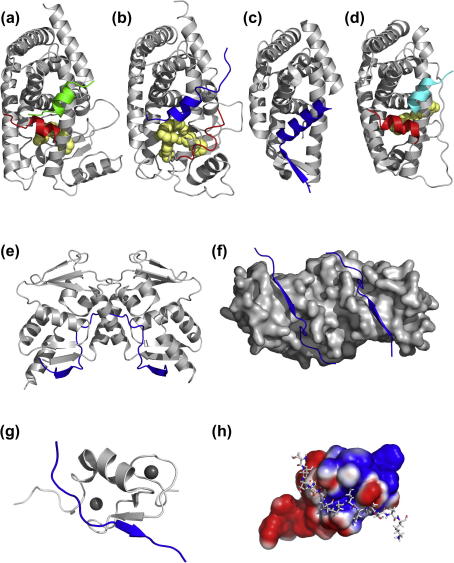
Interaction of transcription factors with co-activator and co-repressor proteins. (a) Structure of the LBD from PPARγ with a peptide from the co-activator SRC1 (shown in green) and the ligand rosiglitazone (yellow) bound (PDB code 2PRG (Nolte et al., 1998)). Helix 12 (red) is in the active position. (b) PPARα LBD structure with the antagonist GW6471 (yellow) and a peptide from ID2 of SMRT bound (blue) (PDB code 1KKQ (Xu et al., 2002)), showing that helix 12 (red) is displaced from the active position. (c) Apo structure of Rev-erbα LBD with a peptide from ID1 of NCoR bound (blue) (PDB code 3N00 (Phelan et al., 2010)). In this structure the co-repressor peptide forms a β-sheet between the N-terminus of the ID1 peptide and the Rev-erbα in addition to the usual α-helix. (d) Structure of the LBD of ERRγ with 4-hydroxytamoxifen (yellow) and a peptide from the co-repressor RIP140 bound (cyan) (PDB code 2GPP (Wang et al., 2006)). The structure shows that the peptide from RIP140 binds in an almost identical manner as the co-activator peptide from SRC1, and helix 12 (red) in the active position. (e and f) Cartoon and surface representations of the structure of the BCL6 BTB domain dimer with peptide from SMRT bound (blue) (PDB code 1R2B (Ahmad et al., 2003)). The peptide from SMRT binds in the lateral groove on BCL6 with the N-terminal amino acids of the SMRT peptide adding a parallel strand to the β1/β5 sheet of BCL6. (g) Structure of the MYND domain of AML1/ETO (gray) with a peptide from SMRT bound (blue), two bound zinc atoms are represented by the dark gray spheres (PDB code 2ODD (Liu et al., 2007)). (h) Electrostatic potential mapped onto a surface representation of the structure of the MYND domain with a peptide from SMRT bound (PDB code 2ODD (Liu et al., 2007)).

As mentioned earlier in Section 1, co-repressors, for the most part, interact with the ligand binding domains (LBDs) of nuclear receptors in the absence of a bound ligand. Much is known about nuclear receptor LBD structure since crystal structures of many such nuclear receptor family LBD domains have now been determined (reviewed in Jin and Yong (2010), Moras D this issue). The overall structure of the LBD is composed of a three-layer antiparallel α-helical sandwich (Fig. 2a). The central layer of helices is incomplete leaving a cavity that serves as the ligand-binding pocket. Bound ligand stabilizes the nuclear receptor conformation through direct contacts with multiple structural elements of the receptor, including helices 3, 5, 6, 7, 10, and the activation helix 12. The LBD structure forms the platform for the recruitment of both co-activator and co-repressor proteins.

In general, nuclear receptor co-activators bind to ligand bound nuclear receptors and co-repressors to un-liganded nuclear receptors (reviewed in McKenna et al. (1999) and Glass and Rosenfeld (2000)). These interactions are mediated by short receptor interaction motifs, multiple copies of which are found within many co-activators and co-repressors. The co-activator interaction motifs conform to the consensus sequence LxxLL with activation dependent on hydrophobic amino-acids in helix 12 (Heery et al., 1997). The co-repressor motif contains a similar amphipathic core to the co-activator motif (φxxφφ where φ is an hydrophobic amino-acid and x any amino-acid but the motif is longer and requires additional flanking sequences (Hu and Lazar, 1999; Nagy et al., 1999; Perissi et al., 1999). SMRT and NCoR have two conserved, co-repressor motifs containing, nuclear receptor interaction domains called ID1 and ID2 (Hu and Lazar, 1999; Nagy et al., 1999; Perissi et al., 1999) (Fig. 1b). Mapping the co-activator and co-repressor binding sites on the surface of the LBD showed that the motifs bind to overlapping surfaces. It was suggested that ligand binding would cause a conformational or dynamic change in helix 12 resulting in displacement of the co-repressor and formation of a suitable co-activator binding surface (Nagy et al., 1999; Perissi et al., 1999).

Within NCoR a third receptor interaction domain has been identified (ID3) (Webb et al., 2000;Cohen et al., 2000). The thyroid hormone receptor (TR) and Rev-erbα nuclear receptors have been shown to interact specifically with the ID3 and ID2 domains of NCoR, with the ID3 being proposed as the major determinant for the interaction of TR with NCoR (Cohen et al., 2000; Webb et al., 2000; Makowski et al., 2003; Astapova et al., 2009; Kim et al., 2010). It has been shown that alternative splicing generates multiple isoforms of SMRT, some of which also include a third receptor interaction domain. Thus splicing may differentially regulate interaction of SMRT with nuclear receptors (Malartre et al., 2004, 2006; Short et al., 2005; Goodson et al., 2005). It has also been suggested that parts of the N- and C-terminal regions of co-repressors, distinct from the “classical” interaction domains, may bind to the DNA binding domain (DBD) of nuclear receptors (Varlakhanova et al., 2010).

Structures of NR LBDs with co-activator peptides bound (PPARγ (Nolte et al., 1998), thyroid receptor (Darimont et al., 1998) and the estrogen receptor (Shiau et al., 1998)) (Fig. 2a) show that the LxxLL co-activator peptide binds to a hydrophobic groove on the surface of the LBD formed by residues from helices 3, 4, 5, and 12 of the LBD. In all cases the co-activator peptide adopts a helical conformation in the crystal structure. The structures also show that interaction of the co-activator peptide requires that helix 12 is in a specific position known as the active position. Helix 12 also adopts this ‘active’ position in several ligand-free structures (PPARγ (Nolte et al., 1998), PXR (Watkins et al., 2001), LRH1 (Sablin et al., 2003)), raising questions as to the exact role of helix 12. Fluorescence anisotropy studies of wild type and mutant forms of PPARγ show that helix 12 is significantly more mobile than the main body of the protein and that upon ligand binding, helix 12 shows reduced mobility, accounting for its role as a sensor of the liganded state of the receptor (Kallenberger et al., 2003).

Crystal structures of antagonist bound LBDs with a peptide from ID2 of SMRT confirmed that the bound co-repressor peptide binds in the same hydrophobic groove as the co-activator, thus rendering co-activator and co-repressor recruitment mutually exclusive. The co-repressor peptide forms three turns of an α-helix, with helix 12 being displaced from the active position (Xu et al., 2002; Wang et al., 2006; Madauss et al., 2007) (Fig. 2b). Two recent structures of LBDs (apo Rev-erbα and RAR with the inverse agonist BMS493) bound to an ID1 peptide from NCoR show a variation in co-repressor binding. In addition to the co-repressor forming an α-helix, there is an anti-parallel β-sheet formed between the N-terminus of the ID1 peptide and the LBD (Phelan et al., 2010; le Maire et al., 2010) (Fig. 2c). Interestingly, the binding of a heme ligand to Rev-erbα promotes the binding of the co-repressor. This is likely to be a consequence of the fact that this receptor lacks helix 12 and does not appear to recruit co-activator proteins.

## 4 Co-repressors recruited to ligand-bound nuclear receptors

The finding that ligand-binding can promote transcriptional repression through the recruitment of co-repressor proteins has turned out to be more prevalent than at first suspected. Perhaps the best understood example is that of RIP140, which acts as a co-repressor, yet interacts with ligand-bound nuclear receptors through multiple co-activator-like LxxLL interaction motifs (Cavaillès et al., 1995; Heery et al., 1997). Thus, in terms of recruitment to receptors, RIP140 acts exactly like a co-activator. However unlike co-activators RIP140 contains four distinct repression domains (Christian et al., 2004), which interact directly with HDACs (Wei et al., 2000), DNA and histone methyltransferases (Kiskinis et al., 2007) and C-terminal binding protein (CtBP) (Christian et al., 2004; Kiskinis et al., 2007; Vo et al., 2001) (Fig. 1a). Like SMRT and NCoR, RIP140 is predicted to be largely intrinsically disordered, containing few regions of predicted structure towards the C-terminus. RIP140 is highly expressed in metabolic tissues and is involved in the regulation of several metabolic processes, including fatty acid and glucose metabolism (reviewed in Fritah et al. (2010)). Intriguingly, like several other co-regulator proteins RIP140 has a dual role since it can serve as a co-activator as well as a co-repressor in liver cells (Herzog et al., 2007).

The three members (α, β and γ) of the Estrogen Related Receptor (ERR) family are constitutively active orphan receptors showing activity in the absence of bound ligand. Structures of the apo form of ERRγ show that the receptor adopts an active conformation in the absence of ligand (Greschik et al., 2002; Wang et al., 2006). Crystal structures have been solved of ERRγ with RIP140 both in the apo form and with a bound agonist (Wang et al., 2006). As would be expected, these structures showed that the LxxLL peptide from RIP140 binds to the receptor in an almost identical manner to the co-activator peptide from SRC1 (in apo and agonist bound structures ERRγ) (Wang et al., 2006) and other nuclear receptor co-activator complexes (Nolte et al., 1998; Darimont et al., 1998; Shiau et al., 1998) (Fig. 2d).

Another co-repressor that is recruited to ligand bound nuclear receptors is LCoR (Ligand Dependent Co-repressor), which is recruited to ligand bound ERα again by means of an LxxLL motif. Like RIP140, LCoR functions by binding directly to the CtBP and to HDACs (Fernandes et al., 2003).

Nuclear receptors and their co-regulatory proteins are subject to various post-translational modifications including phosphorylation, acetylation, SUMOylation, ubiquitinylation and methylation. These modifications act to modulate nuclear receptor activity and their interaction with co-regulators (Wang et al., 2001; Fu et al., 2002; Perissi and Rosenfeld, 2005). Indeed phosphorylation of SMRT/NCoR has been shown to result in redistribution from the nucleus to the cytoplasm and inhibition of their interaction with nuclear receptors (Zhou et al., 2001; Hong and Privalsky, 2000; Baek et al., 2002; Hermanson et al., 2002).

## 5 SMRT/NCoR binding to other transcription factors

SMRT and NCoR interact with a variety of transcription factors in addition to nuclear receptors. In many cases these interactions have only been loosely mapped to broad regions of the co-repressor. However, in a few cases, detailed mapping and structural information about the peptides that mediate these interactions is available (Fig. 1b).

The transcriptional repressor BCL-6 is important for normal B cell development. In B cell lymphomas, structural alterations of the BCL-6 promoter region leads to unregulated expression of BCL-6 and lymphomagenesis (Niu et al., 2003). BCL6 contains a N-terminal BTB domain (identified in the Drosophila melanogaster bric-à-brac, tramtrack and broad complex proteins) and 6 C-terminal zinc fingers, separated by an unstructured central region (Dhordain et al., 1998). Multiple HDACs that in part mediate repression by BCL-6 are recruited to BCL-6 either through direct interactions (Lemercier et al., 2002) or indirectly through the binding of co-repressor complexes to BCL-6 (Wong and Privalsky, 1998; Huynh et al., 2000). The SMRT/NCoR co-repressors and the BCL6 specific co-repressor (BCoR) bind to the BTB domain in a mutually exclusive manner (Dhordain et al., 1998; Melnick et al., 2002; Ahmad et al., 2003; Huynh et al., 2000).

The crystal structure of residues 1414–1430 of SMRT interacting with the BTB domain of BCL6 has been solved (Fig. 2e and f) (Ahmad et al., 2003). The BCL6 BTB domain is an α/β structure and forms a butterfly shaped interwoven obligate homodimer with the N-terminus of one monomer making an anti-parallel β-sheet with the fifth β-strand in the other monomer. The dimer contacts are extensive and include β1, α1, α2, β5 and α6. Two SMRT (1414–1430) peptides bind to the BCL6 BTB dimer in an extended conformation in a groove at the dimer interface. The N-terminal amino-acids of the SMRT peptide add a parallel strand to the β1/β5 sheet. Ser1424 and Ile1425 are deeply buried in a hydrophobic pocket at the BCL6 BTB dimer interface, Ile1428 and His1426 are also buried. The C-terminal amino-acids of the SMRT peptide make contact to the α1 of the other molecule in the dimer. Importantly, the SMRT peptide 1414–1430 is an inhibitor of full-length SMRT binding to BCL-6, and reverses the repressive activities of BCL-6 in vivo (Polo et al., 2004). A peptide inhibitor based on the BCL-6 interaction peptide from SMRT has been shown to be effective against diffuse large B-cell lymphoma (DLBCL) in vitro and in mice models (Cerchietti et al., 2009). This opens the possibility that small molecule inhibitors of the SMRT–BCL-6 interaction might be used therapeutically.

Interestingly, although they both interact with the BCL-6 BTB domain, the interaction peptides from SMRT and BCoR show no sequence similarity. The structure of the BTB domain in complex with a BCoR co-repressor peptide revealed that BCoR binds to the same lateral groove on BCL6 as SMRT thus explaining their mutually exclusive binding (Ghetu et al., 2008). The recruitment of BCoR to BCL-6 has been shown to be involved in the Notch signaling pathway and the repression of Notch target genes (Sakano et al., 2010).

The AML1/ETO chimeric protein results from a t(8;21) chromosome translocation which is found in M2 subtype acute myeloid leukemia (AML) (Miyoshi et al., 1993). AML1/ETO has been shown to interact with several proteins including SMRT/NCoR (Wang et al., 1998; Gelmetti et al., 1998) SIN3 (Amann et al., 2001) and histone deacetylases (Amann et al., 2001). AML1/ETO contains the N-terminal DNA binding domain of AML1 and nearly all of the ETO protein. AML1/ETO contains four Nervy homology regions (NHR) C-terminal to the DNA binding domain. SMRT/NCoR binds to the fourth NHR region (Gelmetti et al., 1998; Lutterbach et al., 1998; Wang et al., 1998) which is also known as the myeloid Nervy DEAF 1 (MYND) domain. Binding is mediated by a NPPPLI motif in SMRT, which is conserved in NCoR (corresponding to residues 1104–1109 of SMRT and residues 1033–1037 of NCoR). A second weaker less well conserved binding site is also present in SMRT and NCoR, corresponding to amino acids 1664–1673 and 606–615, respectively (Liu et al., 2007).

The NMR solution structure of the AML1/ETO MYND domain in complex with a peptide from SMRT (residues 1101–1113) shows that the AML1/ETO MYND domain is structurally homologous to the PHD and RING finger family of proteins, adopting an interleaved zinc-chelating topology and coordinates two zinc atoms (Fig. 2g) (Liu et al., 2007). The peptide from SMRT binds to a hydrophobic pocket in the MYND domain in an extended conformation, with the side chain of Leu1108 from SMRT making a key non-polar contact in a hydrophobic pocket of the MYND domain (Fig. 2h). Other important contacts are made between the side chain of Pro1105 of SMRT packing on top of Trp692 of MYND and hydrogen bonding between the carbonyl oxygen of Pro1106 in SMRT with Q688 of MYND. Leu1108–Ser1110 form a short antiparallel β sheet with Glu672, Thr673 and Cys674 of MYND. Disruption of the interaction of SMRT/NCoR with AML1/ETO though mutations on the MYND domain attenuates the effects of AML1/ETO on cell proliferation, differentiation and gene expression (Liu et al., 2007).

## 6 SMRT/NCoR recruitment of SHARP

In addition to interacting with repressive transcription factors, SMRT and NCoR also mediate interactions with another large co-repressor protein called SHARP. SHARP (SMRT/HDAC1-associated repressor protein) was identified as a component in transcriptional repression complexes recruited by both nuclear receptors and the Notch-signaling factor RBP-Jk (Shi et al., 2001; Oswald et al., 2002). SHARP is a member of the Spen family of proteins, which are characterised by a conserved domain structure of three N-terminal RNA recognition motifs (RRM), and a conserved SPOC (Spen paralog and ortholog C-terminal domain) domain at the C-terminus. The RRM motifs are joined to the SPOC domain by a variable region with low conservation that is predicted to have little or no secondary structure. Contained within this variable region in SHARP is a nuclear receptor interaction domain (RID), that has been shown to mediate binding to nuclear receptors via conserved co-repressor motifs (Shi et al., 2001). The SPOC domain of SHARP was found to bind directly to the negatively charged C-terminus of SMRT/NCoR, which contains a conserved LSD motif (Shi et al., 2001; Ariyoshi and Schwabe, 2003).

**Fig. 3:**
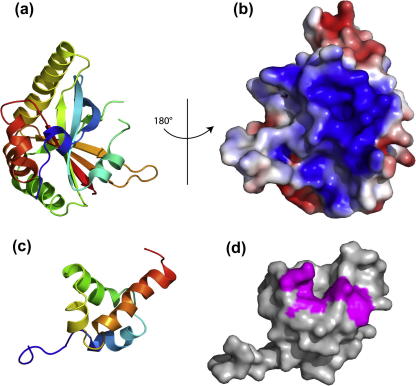
SMRT interaction domain from SHARP and histone deacetylase interaction domain from SMRT. (a) Structure of the SPOC domain from SHARP, showing the seven stranded β-barrel structure framed by seven α-helices (PDB code 1OW1 (Ariyoshi and Schwabe, 2003)). (b) Surface representation of the SPOC domain, the conserved basic patch which mediates binding to SMRT/NCoR is clearly visible. (c and d) Structure and surface representation of the DAD domain (PDB code 1XC5 (Codina et al., 2005)). Residues which when mutated perturb the interaction with HDAC3 are colored magenta on the surface representation.

A crystal structure of the SPOC domain (3495–3664) shows that the structure consists of a seven stranded β-barrel framed by seven α-helices (Fig. 3a and b) (Ariyoshi and Schwabe, 2003). The structure resembles the β-barrel domains of the DNA repair protein Ku80 in the Ku70:Ku80 complex. Mutagenesis and pulldown experiments showed that a conserved basic patch on the surface of the SPOC domain including Tyr3602 binds directly to a 25 amino acid peptide corresponding to a conserved acidic motif at the very C-terminus of SMRT or NCoR (Ariyoshi and Schwabe, 2003) containing the LSD motif (Shi et al., 2001). Although the precise details of this interaction are not known, some clues may be obtained from the packing interactions seen in the crystal. The N-terminus of one SPOC (Pro3495–Gln3500) domain interacts with a β-strand in the basic patch of another (Arg3548–Arg3554). The interactions involve backbone–backbone hydrogen bonds, electrostatic and hydrophobic interactions.

## 7 The core SMRT/NCoR repression complex

**Fig. 4:**
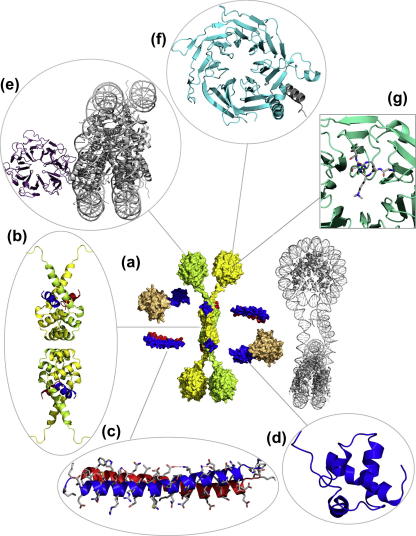
Targeting the core complex to chromatin. Overall model and structural details of the core SMRT/NCoR repression complex. (a) Model of the core SMRT/NCoR repression complex developed from the available structural information, the TBL1–NTD tetramer is shown at the center with the subunits of each dimer colored yellow and green. WD40 domains (PDB code 2H9M (Couture et al., 2006)) are positioned at the C-termini of the TBL1–NTDs. The coiled coil and TBL1 interaction regions from SMRT and GPS2 are shown in blue and red, respectively (PDB code 2XTC (Oberoi et al., 2011)). The SMRT DAD domain (blue) (PDB code 1XC5 (Codina et al., 2005)) is shown next to a HDAC catalytic domain (gold) (PDB code 3HGQ (Lee et al., 2009)). A di-nucleosome (gray) is shown alongside the core complex highlighting the scale of the complex (PDB code 1ZBB (Schalch et al., 2005)). (b) Structural detail of the TBL1 tetramer the subunits of each dimer colored yellow and green, and the interaction regions from SMRT and GPRS are shown in blue and red, respectively (PDB code 2XTC (Oberoi et al., 2011)). (c) Detailed view of the antiparallel coiled coil interaction between SMRT and GPS2 (PDB code 2L5G (Oberoi et al., 2011)). (d) Detailed view of the SMRT DAD domain structure (PDB code 1XC5 (Codina et al., 2005)). (e) Nucleosome interaction with the β-propeller protein RCC1 (PDB code 3MVD (Makde et al., 2010)). (f) RbAp46 β-propeller protein interacting with a helix of histone H4 (PDB code 3CFS (Murzina et al., 2008)). (g) β-Propeller protein EED interacting with a peptide corresponding to trimethylated histone H3K9 (PDB code 3IJ0 (Margueron et al., 2009)).

The interactions discussed so far in this review concern fairly weak and transient, but nevertheless specific, interactions that are made between short peptide motifs in SMRT and NCoR with various transcription factors. The motifs adopt a fixed conformation in the respective complexes, with key residues determining the specificity of the various interactions. In addition to these transient partners, there are also core interaction proteins that appear to be stably associated with the co-repressors and which are essential for the core role of the repressor complexes since they effect the chromatin remodeling functions. These core components interact with a highly conserved amino-terminal region of SMRT that has a strong transcriptional repression activity and has been termed repression domain 1 (RD1). This region contains binding sites for at least three proteins: HDAC3, TBL1/TBLR1 and GPS2. Together with SMRT, or NCoR, these proteins form the core repression complex. The region in SMRT that recruits this core complex, spans amino acids 167–480 and is predicted to be largely unstructured with the exception of a C-terminal helical domain that both recruits and activates HDAC3 and has been termed the deacetylase activation domain (DAD). The amino terminal portion of RD1 recruits both GPS2 and TBL1/TBLR1. Since TBL1 and GPS2 also interact directly with each other, a stable three-way complex is formed. These interactions were originally mapped by Zhang et al. (2002) and refined recently by Oberoi et al. (2011) see Fig. 4.

### 7.1 The SMRT/NCoR deacetylase activation domain

The DAD domain recruits and activates HDAC3 (Guenther et al., 2001; Zhang et al., 2002) and comprises residues 412–480 of SMRT; the first 16-aa form a DAD specific motif and the remaining C-terminal domain forms a SANT-like domain. Truncation of the DAD specific motif abolishes both the interaction with and activation of HDAC3 (Guenther et al., 2001), demonstrating that the SANT-like domain alone is insufficient for HDAC3 interaction and activation (Guenther et al., 2001; Zhang et al., 2002). Other regions within SMRT and NCoR have been reported to recruit HDAC3 but do not activate the enzymatic activity of HDAC3 (Li et al., 2000; Wen et al., 2000; Guenther et al., 2000). The eukaryotic TRiC chaperone complex has been shown to be involved in the assembly of the HDAC3/DAD complex (Guenther et al., 2002) and the activation of HDAC3 has been suggested to be dependent on the phosphorylation of the C-terminal tail of HDAC3 (Zhang et al., 2005).

The NMR structure of the DAD domain (Codina et al., 2005) shows that, whilst the DAD domain has a similar overall fold to the related SANT and MYB domains, it possesses several unique features (Fig. 3c). The DAD domain has an additional helix (0) formed by the second half of the DAD specific domain, the first half of which is unstructured in the NMR structure. The orientations of the three core helices (1, 2 and 3) differ from those within the SANT and MYB domains. Helix 3 of the DAD domain shows the most significant variation in orientation when compared to the other two domains. Importantly, the wider angle of helix 3 in the DAD results in a groove between the amino terminus of helix 3 and the loop between helices 1 and 2. The DAD is rather basic and the groove acquires a basic charge caused by surrounding lysine residues. The hydrophobic potential of the surface shows that whereas both the MYB and SANT domains have mostly polar surfaces, the groove in the DAD has a strikingly high hydrophobic potential, suggesting a binding surface for a non-polar interaction.

Mutational analysis showed that this cleft is involved in the binding and activation of HDAC3 (Fig. 3d). In addition, binding and activation can be decoupled as mutation of Lys449Ala in the DAD domain generates a protein that retains HDAC3 binding but no longer activates (Codina et al., 2005). Somewhat surprisingly, since deletion of the DAD-specific motif abolishes HDAC3 activation, mutations within the unstructured portion of DAD specific domain did not perturb the interaction with HDAC3 or activation of HDAC3. Mutations that disrupt the structure of helix 0 did however result in a loss of HDAC3 activation and binding, suggesting that helix 0 is involved in overall structural stability or in HDAC3 binding (Codina et al., 2005).

The site of interaction with the DAD domain on HDAC3 has been mapped to the N-terminus and C-terminus of HDAC3 and deletion of either of these domains results in the loss of both interaction with the DAD domain and HDAC3 activation (Guenther et al., 2001; Yang et al., 2002). Truncation of the C-terminus also results in the redistribution of HDAC3 from the nucleus to the cytoplasm (Yang et al., 2002). How exactly the binding of HDAC3 to the DAD domain results in activation of HDAC3 remains to be resolved.

### 7.2 A three-way complex between SMRT, GPS2 and TBL1

As mentioned earlier, GPS2 and TBL1 interact with the N-terminal region of the SMRT RD1. In addition to their interaction with SMRT, GPS2 and TBL1 also interact directly with each other. The NMR structure of the interacting regions of SMRT (167–207) and GPS2 (53–90) shows that the interaction is mediated by an anti-parallel coiled-coil (Oberoi et al., 2011) (Fig. 4c). The consequence of the anti-parallel nature of the SMRT/GPS2 complex is that it positions the TBL1 interaction domains of both proteins at one end of the coiled-coil (Fig. 4a).

TBL1 and its closely related homologue TBLR1 (also found to be associated with SMRT and NCoR) have a highly conserved N-terminal domain and a C-terminal WD40 domain. The N-terminal domain of TBL1 contains a LisH domain and mediates the interaction with both GPS2 and SMRT. The crystal structure of the N-terminal domain of TBL1 (residues 1–90) shows that TBL1 is a homotetramer, and would be expected to readily form heterotetramers with TBLR1 (Fig. 4a and b) (Oberoi et al., 2011). The primary homo-dimerisation interface is formed from the conserved LisH domain (6–32) and essentially two helices from each monomer come together to form a four helix bundle. C-terminal to the LisH domain there is an extended and irregular loop followed by a third helix from each monomer. These helices cross and contribute to the dimerisation interface. Two TBL1 dimers interact at the base of the four helix bundle to form the tetramer. This interaction is mediated by interdigitation of surface residues. His23 makes a hydrogen bond with a symmetry related partner and stacks on Phe26. Ile30 is in non-polar contact with Phe26 and Ile30 from another monomer. Tetramerisation in solution was confirmed by mutagenesis of Phe26 and gel filtration (Oberoi et al., 2011).

Mutagenesis experiments show that both GPS2 and SMRT interact with the same hydrophobic groove on TBL1. This groove is formed between the four helix bundle and the third helix. This result seems surprising but can be explained in the context of a heterodimer of GPS2–SMRT binding to a homodimer of TBL1. Since SMRT and GPS2 interact in an anti-parallel fashion this then places the potential interaction helices with TBL1 in opposite orientations meaning that SMRT and GPS2 can make similar interactions with TBL1. A short sequence motif with the consensus A-x-x-A/L-H-R/K-x-Φ (where Φ is a large non-polar residue and x any residue) was identified in both GPS2 and SMRT. Modeling of the interaction of the peptides from GPS2/SMRT with the hydrophobic groove on TBL1 using HADDOCK identified similar interactions for both the GPS2 and SMRT sequences. The common residues that define the interaction motif all make important interactions at the interface. The histidine and non-polar residues are buried at the interface and the basic residue (Arg or Lys) on the back face of the helix interacts with two glutamic acid residues (Glu61 and Glu68) in TBL1. Glu7 (TBL1) is positioned so as to “cap” the SMRT and GPS2 interaction helices, interacting favorably with the helix dipole.

The amino acids involved in dimerisation and tetramerisation of TBL1 are conserved in the closely related protein TBLR1. Hence tetramerisation of TBL1 means that the core co-repressor complex will probably contain four TBL1 or TBLR1 molecules as a scaffold for two GPS2 molecules, two SMRT or NCoR molecules and two HDAC3 molecules. Recent experiments in support of this stoichiometry show that SMRT is able to form homodimers and heterodimers with NCoR (Varlakhanova et al., 2011). This composition for the co-repressor complex would readily account for the 1–2 MDa size observed when purified from nuclear extracts.

TBL1 contains a C-terminal WD40 domain and in this regard is similar to co-repressor proteins such as Sif2p and Tup1 in yeast, Groucho in Drosophila and TLE proteins in humans. PHYRE modeling (Kelley and Sternberg, 2009) of the TBL1 WD40 domain produces a top scoring model where the WD40 domain has seven blades, based on the crystal structure of the WD40 domain from the yeast Tup1 co-repressor (Sprague et al., 2000). The more homologous Sif2p crystal structure (Cerna and Wilson, 2005) also produces a PHYRE model with seven blades for the WD40 domain for TBL1 despite the crystal structure of the Sif2p WD40 domain having eight blades. The human WD40 proteins WDR5 and p55 have been found to interact with histone tails (Couture et al., 2006; Song et al., 2008) and the Drosophila protein RCC1 has been found to interact with nucleosomes (Makde et al., 2010), so it seems probable that the function of the TBL1 WD40 domain may be to interact with chromatin. It is also possible that the WD40 domain might mediate interactions with ubiquitin (Pashkova et al., 2010).

## 8 Perspectives

Transcriptional regulator hub proteins, such as the SMRT and NCoR co-repressors, mediate and integrate interactions with a large number of other factors including transcription factors and enzymes that execute the repressive function of the complex. Structure/function analyses of these proteins and their complexes are challenging due to the largely unstructured nature of these proteins in the absence of their interacting partners. What we have learned to date has relied upon a combination of NMR, crystallographic, mutagenesis and functional assays. Such structural studies are of considerable interest not only to gain insight into the mechanisms through which these enzymatic activities are targeted to chromatin, but also for the potential to develop inhibitor molecules that can specifically inhibit co-repressor interactions with individual transcription factors. A good example of this is the potentially therapeutically useful inhibition of BCL-6 activity through blocking co-repressor recruitment.
